# A Novel Target of Action of Minocycline in NGF-Induced Neurite Outgrowth in PC12 Cells: Translation Initiation Factor eIF4AI

**DOI:** 10.1371/journal.pone.0015430

**Published:** 2010-11-08

**Authors:** Kenji Hashimoto, Tamaki Ishima

**Affiliations:** Division of Clinical Neuroscience, Chiba University Center for Forensic Mental Health, Chiba, Japan; RIKEN Brain Science Institution, Japan

## Abstract

**Background:**

Minocycline, a second-generation tetracycline antibiotic, has potential activity for the treatment of several neurodegenerative and psychiatric disorders. However, its mechanisms of action remain to be determined.

**Methodology/Principal Findings:**

We found that minocycline, but not tetracycline, significantly potentiated nerve growth factor (NGF)-induced neurite outgrowth in PC12 cells, in a concentration dependent manner. Furthermore, we found that the endoplasmic reticulum protein inositol 1,4,5-triphosphate (IP_3_) receptors and several common signaling molecules (PLC-γ, PI3K, Akt, p38 MAPK, c-Jun N-terminal kinase (JNK), mammalian target of rapamycin (mTOR), and Ras/Raf/ERK/MAPK pathways) might be involved in the active mechanism of minocycline. Moreover, we found that a marked increase of the eukaryotic translation initiation factor eIF4AI protein by minocycline, but not tetracycline, might be involved in the active mechanism for NGF-induced neurite outgrowth.

**Conclusions/Significance:**

These findings suggest that eIF4AI might play a role in the novel mechanism of minocycline. Therefore, agents that can increase eIF4AI protein would be novel therapeutic drugs for certain neurodegenerative and psychiatric diseases.

## Introduction

Accumulating evidence suggests that minocycline, a second-generation tetracycline antibiotic, is a potential therapeutic drug for several neurodegenerative and psychiatric disorders [1]–[5]. Minocycline is shown to have beneficial effects in animal models of neurodegenerative disorders, including cerebral ischemia, amyotrophic lateral sclerosis (ALS), Parkinson's disease, Huntington's disease, spinal cord injury, Alzheimer's disease, and multiple sclerosis [6]–[13]. Furthermore, minocycline is also reported to have antipsychotic and neuroprotective effects in animal models of schizophrenia and drug abuse [14]–[18]. A recent double-blind, randomized study demonstrated that minocycline was effective in the treatment of negative and cognitive symptoms of patients with early-phase schizophrenia [19]. In addition, there is a case report showing that minocycline was effective in the treatment of a patient with methamphetamine-related disorders [20]. It is also reported that minocycline reduced craving for cigarettes in humans [21]. Interestingly, minocycline was effective in human immunodeficiency virus (HIV) infection and reactivation as well as HIV-induced neuronal damage, suggesting that this drug has potential as an anti-HIV adjuvant therapy [22], [23]. However, the precise mechanisms underlying the beneficial effects of minocycline are not fully understood.

The PC12 cell, a cell line from the rat pheochromocytoma of the adrenal medulla, is a useful model for studying neurite outgrowth [24], [25]. The purpose of this study is to examine the precise mechanisms underlying the beneficial effects of minocycline. First, we examined the effects of minocycline and two other tetracyclines (tetracycline, doxycycline) on nerve growth factor (NGF)-induced neurite outgrowth in PC12 cells. In this study, we found that minocycline, but not tetracycline, significantly potentiated NGF-induced neurite outgrowth. Second, we examined the precise cellular mechanisms underlying the potentiation by minocycline of NGF-induced neurite outgrowth. Finally, we found that eukaryotic translation initiation factor eIF4AI might be a novel target for the potentiation of NGF-induced neurite outgrowth by minocycline.

## Results

### Effects of three tetracyclines on NGF-induced neurite outgrowth in PC12 cells

**Figure 1:**
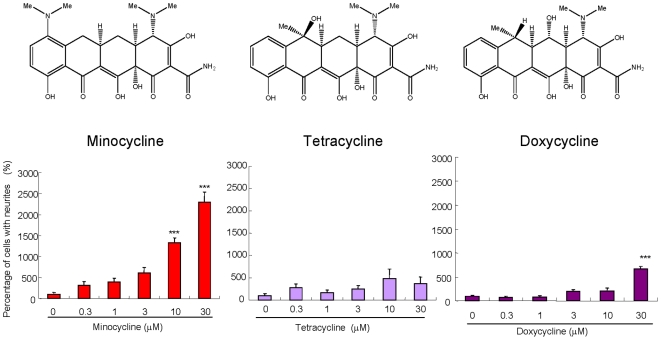
Effects of minocycline, tetracycline, or doxycycline on NGF-induced neurite outgrowth in PC12 cells. Minocycline, but not tetracycline, significantly increased the number of cells with neurite, in a concentration-dependent manner. A high concentration (30 µM) of doxycycline significantly increased the number of cells with neurite. Number is the concentration (µM) of drugs. ***P<0.001 as compared with control (NGF (2.5 ng/ml) alone group). The data show the mean ± SEM (n = 8).

**Figure 2:**
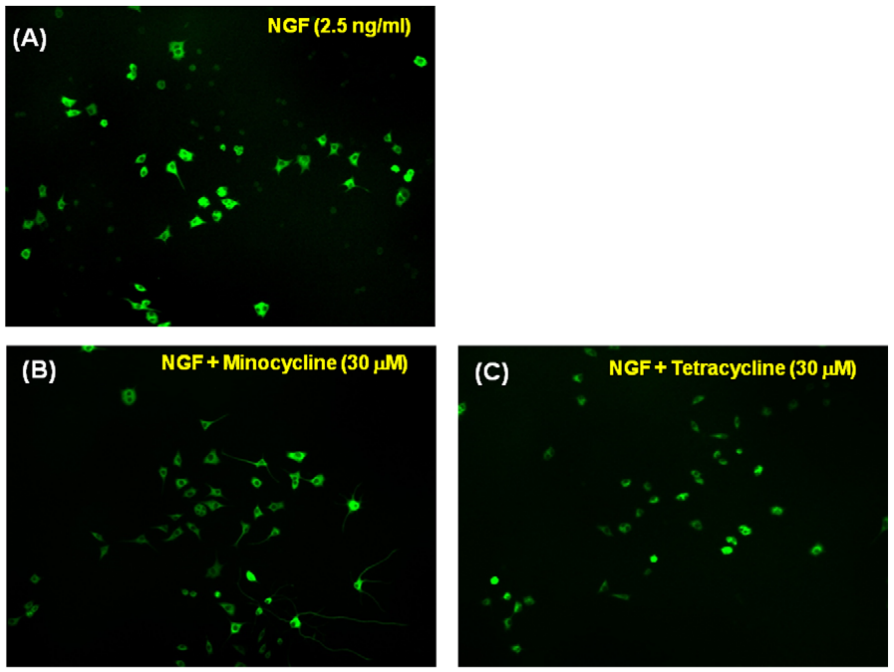
Effects of minocycline and tetracycline on MAP-2 immunocytochemistry in PC12 cells. Representative photographs of MAP-2 immunocytochemistry in PC12 cells. (A) Control (NGF (2.5 ng/ml) alone) (B) NGF + minocycline (30 µM), (C) NGF + tetracycline (30 µM).

Minocycline (0.3, 1.0, 3.0, 10 or 30 µM) significantly increased the number of cells with neurites induced by NGF (2.5 ng/ml), in a concentration-dependent manner (Fig. 1). In contrast, tetracycline (0.3, 1.0, 3.0, 10 or 30 µM) and doxycycline (0.3, 1.0, 3.0, or 10 µM) did not increase the number of cells with NGF (2.5 ng/ml)-induced neurites, although a high concentration of doxycycline (30 µM) significantly increased the number of cells with neurites (Fig. 1). Immunocytochemistry using microtubule-associated protein 2 (MAP-2) antibody showed that minocycline (30 µM), but not tetracycline (30 µM), increased the MAP-2 immunoreactivity in the cells with neurite (Fig. 2).

### Role of signaling molecules proximal to TrkA in the potentiation of NGF-induced neurite outgrowth by minocycline

**Figure 3:**
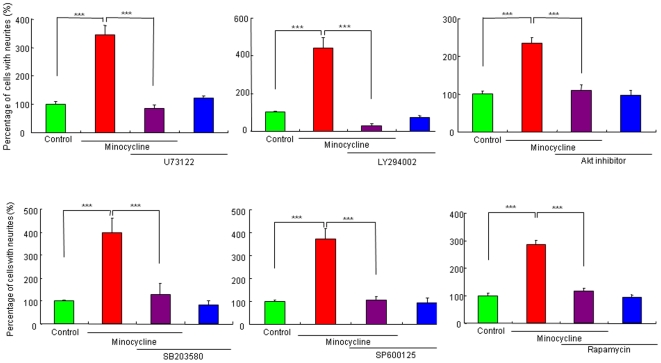
Effects of the specific inhibitors of PLC-γ, PI3K, p38MAPK, JNK, and mTOR on potentiation of NGF-induced neurite outgrowth by minocycline. The potentiating effects of minocycline (30 µM) on the NGF (2.5 ng/ml)-induced neurite outgrowth were antagonized by co-administration of the PLC-γ inhibitor (U73122; 1.0 µM), the PI3K inhibitor (LY294002; 10 µM), Akt inhibitor (1.0 µM), the p38MAPK inhibitor (SB203580; 10 µM), the JNK inhibitor (SP600125; 10 µM), and mTOR inhibitor (rapamycin; 5.0 µM). ***P<0.001 as compared with control (NGF (2.5 ng/ml) alone group). The data show the mean ± SEM (n = 6–18).

We examined the effects of the specific inhibitors of PLC-γ, PI3K, Akt, p38 MAPK, c-Jun N-terminal kinase (JNK) and mammalian target of rapamycin (mTOR), since these signaling molecules are activated upon the addition of NGF [24], [26]–[28]. The PLC-γ inhibitor (U73122; 1.0 µM), PI3K inhibitor (LY294002; 10 µM), Akt inhibitor (1.0 µM), p38 MAPK inhibitor (SB203580; 10 µM), JNK inhibitor (SP600125; 10 µM), and mTOR inhibitor (rapamycin; 5.0 µM) significantly blocked the potentiation of NGF-induced neurite outgrowth by minocycline (30 µM) (Fig. 3). In contrast, these inhibitors alone did not alter NGF-induced neurite outgrowth in PC12 cells (Fig. 3).

### Role of the Ras/Raf/ERK/MAPK pathway in the potentiation of NGF-induced neurite outgrowth by minocycline

**Figure 4:**
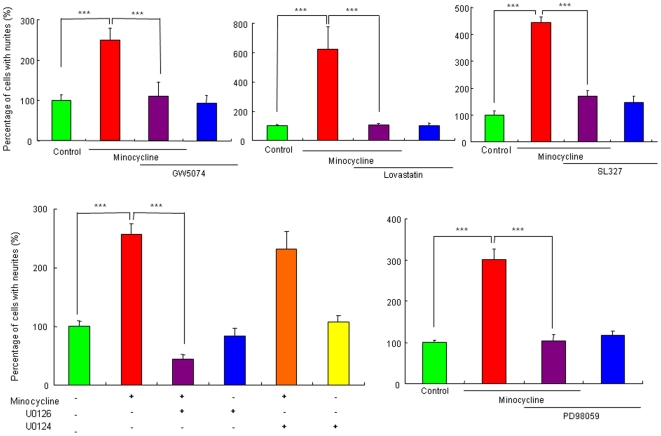
Effects of the specific inhibitors of Ras, Raf, MEK1/2, and MAPK on potentiation of NGF-induced neurite outgrowth by minocycline. The potentiating effects of minocycline (30 µM) on the NGF-induced neurite outgrowth were antagonized by co-administration of the Ras inhibitor (GW5074; 1.0 µM), the Raf inhibitor (lovastatin; 10 µM), the MEK inhibitor (U0126; 10 µM), the MEK1/2 inhibitor (SL327; 10 µM),and the MAPK inhibitor (PD98059; 10 µM). In contrast, U0124 (10 µM), an inactive analog of U0126, did not alter the number of cells with neurite by minocycline treatment. ***P<0.001 as compared with control (NGF (2.5 ng/ml) alone group). The data show the mean ± SEM (n = 6–14).

The Ras/Raf/ERK/MAPK pathway is known to be involved in NGF-induced neurite outgrowth [24], [26], [27]. Therefore, we examined the effects of this pathway's specific inhibitors. The Ras inhibitor (GW5074; 1.0 µM), Raf inhibitor (lovastatin; 10 µM), MEK inhibitor (U0126; 10 µM), MEK1/2 inhibitor (SL327; 10 µM), and MAPK inhibitor (PD98059; 10 µM) significantly blocked the potentiation of NGF-induced neurite outgrowth by minocycline (30 µM) (Fig. 4). In contrast, U0124 (10 µM), an inactive analog of U0126, did not alter the potentiation of NGF-induced neurite outgrowth by minocycline. Furthermore, these inhibitors alone did not alter NGF-induced neurite outgrowth in PC12 cells (Fig. 4).

### Role of IP_3_ receptors in the potentiation of NGF-induced neurite outgrowth by minocycline

**Figure 5:**
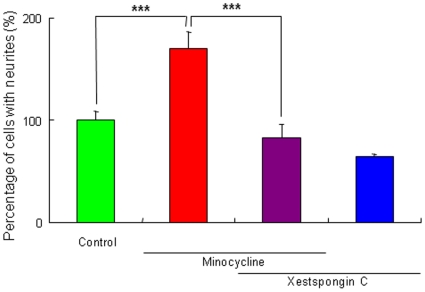
Effects of the IP_3_ receptor antagonist on potentiation of NGF-induced neurite outgrowth by minocycline. The potentiating effects of minocycline (30 µM) on the NGF-induced neurite outgrowth were antagonized by co-administration of the selective IP_3_ receptor antagonist xestspongin C (1.0 µM). In contrast, xestspongin C (1.0 µM) alone did not alter NGF-induced neurite outgrowth. ***P<0.001 as compared with control (NGF (2.5 ng/ml) alone group). The data show the mean ± SEM (n = 6–18).

Previously, we reported that receptors of the endoplasmic reticulum (ER) protein inositol 1,4,5-triphosphate (IP_3_) play a role in the potentiation of NGF-induced neurite outgrowth by the antidepressant fluvoxamine [24] and anti-dementia drug donepezil [25]. To investigate the role of IP_3_ receptors in minocycline's action on NGF-induced neurite outgrowth, we examined the effects of xestospongin C (a selective, reversible, and membrane-permeable inhibitor of IP_3_ receptors) [29] on the effects of minocycline on NGF-induced neurite outgrowth. Co-administration of xestospongin C (1.0 µM) significantly blocked the potentiation of NGF-induced neurite outgrowth by minocycline (30 µM) (Fig. 5). Furthermore, administration of xestospongin C (1.0 µM) alone did not alter NGF-induced neurite outgrowth in PC12 cells (Fig. 5).

### Lack of PARP-1 and 5-lipooxygenase in the potentiation of NGF-induced neurite outgrowth by minocycline

**Figure 6:**
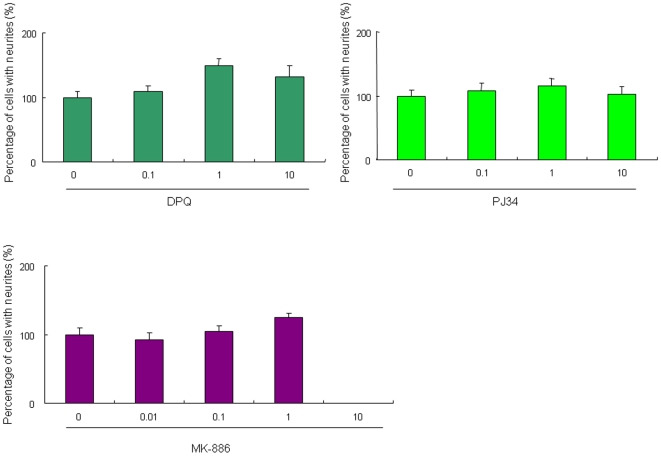
Effects of PARP-1 inhibitors and 5-lipoxygenase inhibitor on NGF-induced neurite outgrowth in PC12 cells. Administration of PARP-1 inhibitors (DPQ or PJ34) or selective 5-lipoxygenase inhibitor MK-886 did not alter the NGF-induced neurite outgrowth in PC12 cells. The data show the mean ± SEM (n = 6–18).

It has been reported that minocycline is a potent inhibitor for poly (ADP-ribose) polymerase-1 (PARP-1), which is involved in neuroprotective actions [30]. To investigate the role of PARP-1, we examined the effects of two established PARP-1 inhibitors, DPQ [3,4-dihydro-5[4-(1-piperindinyl)butoxy]-1(2*H*)-isoquinoline] and PJ34 [*N*-(6-oxo-5, 6-dihydrophenanthridin-2-yl)-*N,N*-dimethylacetamide hydrochloride], on the NGF-induced neurite outgrowth in PC12 cells. Administration of DPQ (0.1, 1 and 10 µM) or PJ34 (0.1, 1 and 10 µM) did not alter the NGF-induced neurite outgrowth (Fig. 6).

Furthermore, minocycline has been reported to protect against N-methyl-D-aspartate (NMDA)-induced neuronal injury via inhibiting 5-lipoxygenase activation, which is involved in the neuroprotective actions [31]. To investigate the role of 5-lipoxygenase, we examined the effects of MK-886, the established inhibitor of 5-lipoxygenase-activation protein, on the potentiation of NGF-induced neurite outgrowth by minocycline. Administration of MK-886 (0.01, 0.1, and 1 µM) did not alter the NGF-induced neurite outgrowth in PC12 cells (Fig. 6). In contrast, a high concentration of MK-886 (10 µM) significantly decreased the number of PC12 cells with neurite outgrowth (Fig. 6), suggesting that a high concentration (10 µM) of MK-886 may cause cytotoxicity in PC12 cells.

### Role of eIF4AI in the potentiation of NGF-induced neurite outgrowth by minocycline

To determine the molecular target of minocycline's action on NGF-induced neurite outgrowth, we performed two-dimensional gel electrophoresis proteome analysis. In PC12 cells, we found increased levels of eukaryotic translation initiation factor eIF4AI protein, an RNA-dependent ATPase and an ATP-dependent helicase that unwinds the local secondary structure in mRNA to allow binding of the 43S ribosomal complex [32]–[34], after treatment with minocycline (30 µM) but not tetracycline (30 µM) (Fig. S1).

**Figure 7:**
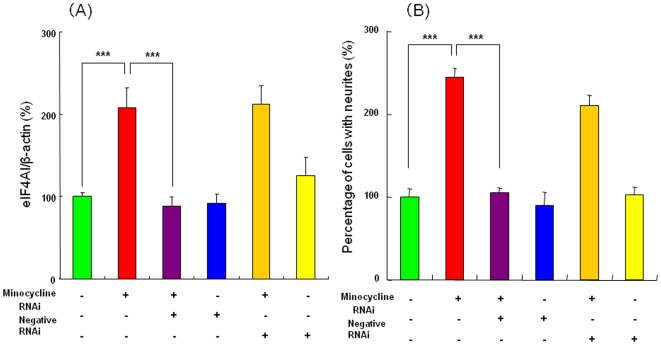
Increase in eIF4AI protein is required for minocycline-induced potentiation of NGF-induced neurite outgrowth in PC12 cells. (A) The potentiating effects of minocycline (30 µM) on the eIF4AI protein levels were significantly antagonized by treatment of eIF4AI RNAi, but not negative RNAi. In contrast, eIF4AI RNAi or negative RNAi alone did not alter the levels of eIF4AI protein in the control (NGF (2.5 ng/ml)-treated) group. The data show the mean ± SEM (n = 8). ***p<0.001 as compared with minocycline (30 µM) group. (B) The potentiating effects of minocycline (30 µM) on the NGF-induced neurite outgrowth were significantly antagonized by treatment of eIF4AI RNAi, but not negative RNAi. In contrast, eIF4AI RNAi or negative RNAi alone did not alter NGF (2.5 ng/ml)-induced neurite outgrowth. The data show the mean ± SEM (n = 8). ***p<0.001 as compared with minocycline (30 µM) group.

To determine whether eIF4AI mediates the potentiation of NGF-induced neurite outgrowth by minocycline, we treated PC12 cells with eIF4AI RNA interference (RNAi), which reduces the expression of the eIF4AI protein. As shown in Fig. 7A, the increase of the eIF4AI protein by minocycline (30 µM) was significantly blocked by treatment with eIF4AI RNAi, but not by the negative control of eIF4AI RNAi. In contrast, neither treatment with eIF4AI RNAi nor that by the negative control of eIF4AI RNAi altered the basal levels of eIF4AI protein (Fig. 7A). Furthermore, the potentiating effects of minocycline on NGF-induced neurite outgrowth were significantly antagonized by treatment with eIF4AI RNAi, but not by the negative control of eIF4AI (Fig. 7B). In contrast, neither treatment with eIF4AI RNAi nor that with the negative control of eIF4AI RNAi altered the NGF-induced neurite outgrowth in PC12 cells (Fig. 7B).

## Discussion

The major findings of this study are that minocycline, but not tetracycline, could potentiate NGF-induced neurite outgrowth in PC12 cells, and that the IP_3_ receptors and several common cellular signaling pathways might be involved in the mechanism of action for potentiation of NGF-induced neurite outgrowth by minocycline. Interestingly, we identified the eukaryotic initiation factor eIF4AI as distinguishing different protein levels of minocycline and tetracycline. This study suggests that increase in the eIF4AI protein by minocycline might contribute to the potentiation of NGF-induced neurite outgrowth by this drug. Therefore, it is likely that eIF4AI is a novel cellular target for minocycline's action.

NGF binds to the high-affinity tyrosine receptor TrkA, initiating several signaling pathways affecting both morphological and transcriptional targets [24], [26], [27]. The signaling molecules, including PLC-γ, PI3K, Akt, p38 MAPK, and JNK, are activated upon the addition of NGF [35]. PLC-γ catalyzes the hydrolysis of phosphatidylinositol-4,5-bisphosphate (PIP_2_) to diacylglycerol (DAG) and IP_3_. DAG activates protein kinase C, and IP_3_ promotes transient release of Ca^2+^ from the ER via stimulation at IP_3_ receptors. Thus, the pathway via PLC-γ is responsible for NGF-induced neurite outgrowth [24], [36]. Furthermore, stimulation of PI3K is reported to be involved in the promotion of neurite outgrowth in PC12 cells [24], [37]. In this study, we found that the PLC-γ inhibitor U73122, the PI3K inhibitor LY294002, the Akt inhibitor, and the mTOR inhibitor rapamycin significantly blocked the potentiation of NGF-induced neurite outgrowth by minocycline. Moreover, we found that both the p38MAPK inhibitor SB203580 and the JNK inhibitor SP600125 significantly blocked the potentiation of NGF-induced neurite outgrowth by minocycline. Additionally, we found that the specific inhibitors for the Ras/Raf/MEK/MAPK pathways significantly blocked the potentiation of NGF-induced neurite outgrowth by minocycline. Taken together, these findings suggest that common signaling pathways, including PLC-γ, PI3K, Akt-mTOR, p38MAPK, the JNK, and the Ras/Raf/MEK/MAPK, are involved in the mechanisms of the potentiation of NGF-induced neurite outgrowth by minocycline. Considering the role of the PI3K/Akt/mTOR pathway and the ERK/MAPK signaling pathway in the control of protein synthesis-dependent learning and memory [38], the present results may be of interest.

Several clinical studies showed that a single subanaesthetic dose of the N-methyl-D-aspartate (NMDA) receptor antagonist ketamine caused a rapid antidepressant effect within hours of administration in treatment-refractory patients with major depression [39], [40]. Accumulating evidence suggests that the excitatory amino acid glutamate plays a key role in the pathophysiology of major depression although the precise mechanisms underlying rapid-antidepressant effects of ketamine are unclear [41]–[43]. Very recently, Li et al. [44] reported the role of the mTOR signaling pathway in the rapid antidepressant effects of ketamine. Ketamine rapidly activated the mTOR signaling pathway, leading to increased synaptic signaling proteins and increased number and function of new spines synapses in the rat prefrontal cortex. Infusion of the mTOR inhibitor rapamycin into rat prefrontal cortex prevented antidepressant-like effects of ketamine in several animal models. Activation of mTOR signaling caused increased levels of the phosphorylated and activated forms of eukaryotic initiation factor 4E binding protein (4E-BP1). This paper suggests that the rapid activation of mTOR signaling pathway may be an important role in the mechanisms of rapid antidepressant effects of the NMDA receptor antagonists [44] since PI3K/Akt/mTOR signaling pathway are involved in the neurite outgrowth and the control of protein synthesis-dependent learning and memory [38], [45], [46]. Interestingly, there is a case reporting that minocycline was effective in the treatment of depressive symptoms in a patient with mood disorder [47], suggesting a possible antidepressant effect of minocycline [48]. Taken together, it is likely that minocycline might have antidepressant-activity since Akt/mTOR signaling pathway is involved in the mechanisms of potentiation of NGF-induced neurite outgrowth by this drug.

The intracellular Ca^2+^ is an important regulator of neurite outgrowth [49], [50]. It has been reported that calcium signaling mediated by IP_3_ receptors resulted in neurite outgrowth, suggesting that IP_3_-mediated Ca^2+^ release from internal stores is necessary to maintain [Ca^2+^]_i_, within the optimum range of neurite outgrowth [51]. In the present study, we found that the IP_3_ receptor antagonist xestospongin C significantly blocked the potentiation of NGF-induced neurite outgrowth by minocycline, suggesting the role of IP_3_ receptors on the potentiation of NGF-induced neurite outgrowth by minocycline. Previously, we reported that IP_3_ receptors play a role in the potentiation of NGF-induced neurite outgrowth by other drugs, including fluvoxamine, donepezil, or ROCK inhibitor Y-27632 [24], [25], [52]. Taken together, it seems that the IP_3_ receptors on the ER play an important role in the mechanism underlying the potentiation of NGF-induced neurite outgrowth by minocycline.

Protein synthesis (or translation) in eukaryotic cells is fundamental for gene expression and is mainly regulated at the initiation step. Translation initiation is a complex process that begins with interaction of the cap-binding protein complex eukaryotic initiation factor 4 (eIF4) families. First, eIF4E binds the cap structure at the 5′-untranslated region (UTR) of the mRNA. Next, eIF4A, an ATPase/RNA helicase, unwinds the secondary structure in the 5′-UTR, allowing the small ribosomal subunit to scan along the mRNA and to reach the start codon [34], [53]. In the present study, we found that an increase in the levels of eIF4AI protein by minocycline might play a role in the mechanism of the potentiation of NGF-induced neurite outgrowth by minocycline although the precise mechanisms underlying the minocycline-induced increase of eIF4AI are currently unclear. Very recently, Fukao et al. [54] reported that the RNA-binding protein HuD can induce neurite outgrowth in PC12 cells through direct interaction with eIF4A in the 5′ cap-binding complex, suggesting the important role of eIF4A in neuronal outgrowth [54], [55]. Taken together, it is likely that eIF4A families including eIF4AI play a role in neurite outgrowth, indicating that eIF4A may be a potential target for developing therapeutic drugs for neurodegenerative and psychiatric diseases. Therefore, agents that can increase the eIF4A protein may have therapeutic relevance in diverse conditions with altered neurite outgrowth.

Translation initiation factors have been implicated in the pathophysiology of certain neuropsychiatric diseases because translational control plays a role in neuronal plasticity [38], [56]. It is also suggested that dysregulation of the translational control might play a part in cancer or the neurodegenerative disease termed “vanishing white matter” [57], [58]. Thus, it seems that mutations or reduced expression of translation initiation factors might be implicated in the pathophysiology of several diseases. Considering the beneficial effects of minocycline in several animal models, it is likely that eukaryotic translation initiation factors, including eIF4AI, would be novel therapeutic targets for certain neurodegenerative and psychiatric diseases.

Postmortem human brains are critical for examining molecular changes associated with the pathophysiology of neuropsychiatric diseases. At present, there are no reports showing alteration in translation initiation factors eIF4AI in the postmortem brain samples from patients with neuropsychiatric diseases. However, there is a paper reporting that the strong expression of phosphorylation of another translation initiation factor eIF2α observed in subpopulations of neurons bearing neurofibrillary tangles or pretangles in the postmortem brain from patients with Alzheimer's disease [59], suggesting that factors linked with tau deposition might regulate protein synthesis throughout eIF2α phosphorylation in certain neurons of Alzheimer's disease. Therefore, it may be interesting to examine whether expression of eIF4AI is altered in the postmortem brain samples from neuropsychiatric disease.

Recently, Bruno et al. [60] reported that, in a transgenic mouse Alzheimer's disease model, minocycline could diminish altered matrix metalloproteinase 9, an enzyme of NGF degradation, suggesting that minocycline may affect extracellular concentration of NGF in the cell culture. Using a NGF immunoassay system, we measured the levels of NGF in the cell culture medium in order to examine whether minocycline can affect NGF concentration in the medium of PC12 cells. However, we did not find any change of NGF levels in the cell culture treated with vehicle or minocycline (Fig. S2). Therefore, it is unlikely that minocycline alter the stability/activity of added NGF in the PC12 cells.

In conclusion, the present results suggest that minocycline, but not tetracycline, could potentiate NGF-induced neurite outgrowth in PC12 cells, and that interaction with IP_3_ receptors and several cellular signaling pathways are involved in the mechanism underlying the pharmacological action of minocycline. Furthermore, we identified eIF4AI as a novel target for the mechanisms of action of minocycline. Finally, these findings offer new approaches for developing potential therapeutic drugs that can target translation initiation factors including eIF4A.

## Materials and Methods

### Drugs

The drugs were obtained from the following sources: xestospongin C, MK-886 (Wako Pure Chemicals Inc., Tokyo, Japan); minocycline hydrochloride, doxycycline hydrochloride, tetracycline hydrochloride, LY294002 (Sigma-Aldrich, St Louis, MO); NGF (Promega, Madison, WI); lovastatin, PD98059, GW5074, SB203580, MEK 1/2 inhibitor (SL327), SP600125, U0126, U0124, DPQ, PJ34, rapamycin (Calbiochem-Novabiochem, San Diego, CA), and Akt inhibitor (Bio Vision Inc., CA). Other drugs were purchased from commercial sources.

### Cell culture

PC12 sells (RIKEN Cell Bank, Tsukuba, Japan) were cultured at 37°C, 5% CO_2_ with Dulbecco's modified Eagle's medium (DMEM) supplemented with 5% heat-inactivated fetal bovine serum (FBS), 10% heat-inactivated horse serum, and 1% penicillin. The medium was changed two or three times a week. PC12 cells were plated onto 24-well tissue culture plates coated with poly-D-lysine/laminin. Cells were plated at relatively low density (0.25×10^4^ cells/cm^2^) in DMEM medium containing 0.5% FBS, 1% penicillin streptomycin. Medium containing a minimal level of serum (0.5% FBS) was used as previously reported [24], [25]. Previously, we examined the optimal concentration of NGF for NGF-induced neurite outgrowth in PC12 cells. NGF (2.5, 5, 10, 20, 40 ng/ml) increased the number of cells with neurite outgrowth in PC12 cells, in a concentration-dependent manner [24]. In the present studies, 2.5 ng/ml of NGF was used to study the potentiating effects of tetracyclines on NGF-induced neurite outgrowth. Twenty-four hours after plating, the medium was replaced with DMEM medium containing 0.5% FBS and 1% penicillin streptomycin with NGF (2.5 ng/ml) with or without several drugs.

### Quantification of neurite sprouting

Four days after incubation with NGF (2.5 ng/ml) with or without the several drugs, morphometric analysis was performed on digitized images of live cells taken under phase-contrast illumination with an inverted microscope linked to a camera. Images of three fields per well were taken, with an average of 100 cells per field. Differentiated cells were counted by visual examination of the field; only cells that had at least one neurite with a length equal to the cell body diameter were counted, and were then expressed as a percentage of the total cells in the field. The counting was performed in a blinded manner.

### MAP-2 immunocytochemistry in PC12 cells

Cells were fixed for 30 min at room temperature with 4% paraformaldehyde then permeabilized with 0.2% Triton and blocked with 1.5% normal goat serum, 0.1% bovine serum albumin (BSA) in 0.1 M phosphate-buffer saline for 1 h to reduce nonspecific binding. Cells were incubated overnight at 4°C with anti-microtubule-associated protein 2 (MAP-2) antibodies (1∶1000 dilution in blocking solution, Chemicon International, Temecula, CA, USA). The immunolabeling was visualized with secondary antibodies conjugated to Alexa-488 (1∶1000; Invitrogen, Carlsbad, CA, USA). MAP-2 immuncytochemistry was visualized with a fluorescence microscope (Axiovert 200, Carl Zeiss, Oberkocken, Germany).

### Western blot analysis

PC12 cells were washed with PBS and lysed in Laemmli lysis buffer. Aliquots (30 µg) of the proteins were measured by DC protein assay kit (Bio-Rad, Hercules, CA, USA) and incubated for 5 min at 95°C with an equal volume of 125 mM Tris/HCl, pH 6.8, 20% glycerol, 0.1% bromphenol blue, 10% β-mercaptoethanol, 4% SDS, and subjected to SDS-PAGE using 7.5% mini-gels (Mini ProteanII; Bio-Rad, Hercules, CA, USA). Proteins were transferred onto PVDF membranes using a Trans Blot Mini Cell (Bio-Rad, Hercules, CA, USA). For immunodetection, the blots were blocked for 1 h in TBST (50 mM Tris/HCl, pH 7.8, 0.13 M NaCl, 0.1% Tween 20) containing 5% nonfat dry milk at room temperature (RT), followed by incubation with rabbit anti-eIF4AI antibody (1∶250, ab31217, Abcam, Cambridge, UK) overnight at 4°C in TBST/5% blocker. The blots were washed five times with TBST. Incubation with the secondary antibody (GE Healthcare Bioscience, UK) was performed for 1 h at RT. After extensive washing, immunoreactivity was detected by ECL plus Western Blotting Detection system (GE Healthcare Bioscience, UK). Images were captured using a Fuji LAS3000-mini imaging system (Fujifilm, Tokyo, Japan) with the Multi Gauge software (Ver.3.0; Fujifilm, Tokyo, Japan) and immunoreactive bands were quantified. β-actin immunoreactivity was used to monitor equal sample loading.

### RNAi transfection

RNAi gene expression knockdown studies were performed using the TriFECTa RNAi kit (Integrated DNA Technologies, Coralville, CA) and corresponding protocol. Each 27 mer RNAi duplex was transfected into cells using Lipofectamine 2000 reagent (Invitrogen, Carlsbad, CA) following the manufacturer's guidelines. RNAi was purchased from Integrated DNA Technologies (Coralville, CA). The following sequences: Rattus norvegicus eukaryotic translation initiation factor 4AI (Eif4aI), mRNA GenBank Accession No. NM_199372 (RNC.RNAI.N199372.10.1; IDT): sense, 5′-GGAACGAGACGUGAUCAUGAGGGAG-3′; antisense, 5′-CUCCCUCAUGAUCACGUCUCGUUCCUU-3′ (RNC.RNAI.N199372.10.2; IDT): sense, 5′-CCUAAUCACCUCAUUCCUAAAGGCT-3′; antisense, 5′-AGCCUUUAGGAAUGAGGUGAUUAGGUU-3′(RNC.RNAI.N199372.10.3; IDT): sense, 5′-GCUGGACCAAAUCUACAGGGAGAAC-3′; antisense, 5′-GUUCUCCCUGUAGAUUUGGUCCAGCAA-3′. For all relative control experiments, cells were exposed to a scrambled non-specific RNAi duplex with the following sequence: sense, 5′-CUUCCUCUCUUUCUCUCCCUUGUGA-3′; antisense, 5′-UCACAAGGGAGAGAAAGAGAGGAAGGA-3′.

### Statistical analysis

Data are expressed as means ± standard error of the mean (SEM). Statistical analysis was performed by using one-way analysis of variance (ANOVA) and the *post hoc* Bonferroni/Dunn test. *P* values less than 0.05 were considered statistically significant.

## Supporting Information

Figure S1 **Effects of minocycline and tetracycline on eIF4AI levels in PC12 cells** PC12 cells were treated with control (NGF (2.5 ng/ml)), minocycline (30 µM) or tetracycline (30 µM) for 5 days. Then cells were washed with PBS, and lysed in Laemmli lysis buffer. Western blot analysis was performed using rabbit anti-eIF4AI antibody (1∶250, ab31217, Abcam, Cambridge, UK). The levels of eIF4AI protein in PC12 cells were significantly increased by treatment with minocycline (30 µM), but not tetracycline (30 µM). The data show the mean ± SEM (n = 8). ***p<0.001 as compared with control (NGF (2.5 ng/ml) alone) group. (TIF)

Figure S2 **Effects of minocycline on NGF levels in PC12 cells**
PC12 cells were treated with control (NGF (2.5 ng/ml)) or minocycline (30 µM) for 5 days. The levels of NGF in the culture medium were measured using NGF E_max_ Immunoassay system (Promega, Madison, WI). The levels of NGF in the culture medium of PC12 cells were not altered by treatment with minocycline (30 µM). The data show the mean ± SEM (n = 6). (DOC)

## References

[1] Domercq M, Matute C (2004). Neuroprotection by tetracyclines.. Trends Pharmacol Sci.

[2] Blum D, Chtarto A, Tenenbaum L, Brotchi J, Levivier M (2004). Clinical potential of minocycline for neurodegenerative disorders.. Neurobiol Dis.

[3] Thomas M, Le WD (2004). Minocycline: neuroprotective mechanisms in Parkinson's disease.. Curr Pharm Des.

[4] Miyaoka T (2008). Clinical potential of minocycline for schizophrenia.. CNS Neurol Disord Drug Targets.

[5] Orsucci D, Calsolaro V, Mancuso M, Siciliano G (2009). Neuroprotective effects of tetracyclines: molecular targets, animal models and human disease.. CNS Neurol Disord Drug Targets.

[6] Yrjänheikki J, Tikka T, Keinänen R, Goldsteins G, Chan PH (1999). A tetracycline derivative, minocycline, reduces inflammation and protects against focal cerebral ischemia with a wide therapeutic window.. Proc Natl Acad Sci USA.

[7] Kriz J, Nguyen MD, Julien JP (2002). Minocycline slows disease progression in a mouse model of amyotrophic lateral sclerosis.. Neurobiol Dis.

[8] Du Y, Ma Z, Lin S, Dodel RC, Gao F (2001). Minocycline prevents nigrostriatal dopaminergic neurodegeneration in the MPTP model of Parkinson's disease.. Proc Natl Acad Sci USA.

[9] Wang X, Zhu S, Drozda M, Zhang W, Stavrovskaya IG (2003). Minocycline inhibits caspase-independent and -dependent mitochondrial cell death pathways in models of Huntington's disease.. Proc Natl Acad Sci USA.

[10] Menalled LB, Patry M, Ragland N, Lowden PA, Goodman J (2010). Comprehensive behavioral testing in the R6/2 mouse model of Huntington's disease shows no benefit from CoQ10 or minocycline.. PLoS One.

[11] Teng YD, Choi H, Onario RC, Zhu S, Desilets FC (2004). Minocycline inhibits contusion-triggered mitochondrial cytochrome c release and mitigates functional deficits after spinal cord injury.. Proc Natl Acad Sci USA.

[12] Choi Y, Kim HS, Shin KY, Kim EM, Kim M (2007). Minocycline attenuates neuronal cell death and improves cognitive impairment in Alzheimer's disease models.. Neuropsychopharmacology.

[13] Hu W, Metselaar J, Ben LH, Cravens PD, Singh MP (2009). PEG minocycline-liposomes ameliorate CNS autoimmune disease.. PLoS One.

[14] Zhang L, Kitaichi K, Fujimoto Y, Nakayama H, Shimizu E (2006). Protective effects of minocycline on behavioral changes and neurotoxicity in mice after administration of methamphetamine.. Prog Neuropsychopharmacol Biol Psychiatry.

[15] Zhang L, Shirayama Y, Iyo M, Hashimoto K (2007). Minocycline attenuates hyperlocomotion and prepulse inhibition deficits in mice after administration of the NMDA receptor antagonist dizocilpine.. Neuropsychopharmacology.

[16] Levkovitz Y, Levi U, Braw Y, Cohen H (2007). Minocycline, a second-generation tetracycline, as a neuroprotective agent in an animal model of schizophrenia.. Brain Res.

[17] Hashimoto K, Tsukada H, Nishiyama S, Fukumoto D, Kakiuchi T (2007). Protective effects of minocycline on the reduction of dopamine transporters in the striatum after administration of methamphetamine: a positron emission tomography study in conscious monkeys.. Biol Psychiatry.

[18] Fujita Y, Ishima T, Kunitachi S, Hagiwara H, Zhang L (2006). Phencyclidine-induced cognitive deficits in mice are improved by subsequent subchronic administration of the antibiotic drug minocycline.. Prog Neuropsychopharmacol Biol Psychiatry.

[19] Levkovitz Y, Mendlovich S, Riwkes S, Braw Y, Levkovitch-Verbin H (2010). A double-blind, randomized study of minocycline for the treatment of negative and cognitive symptoms in early-phase schizophrenia.. J Clin Psychiatry.

[20] Tanibuchi Y, Shimagami M, Fukami G, Sekine Y, Iyo M (2010). A case of methamphetamine use disorder treated with the antibiotic drug minocycline.. Gen Hosp Psychiatry in press.

[21] Sofuoglu M, Waters AJ, Mooney M, O'Malley SS (2010). Minocycline reduced craving for cigarettes but did not affect smoking or intravenous nicotine responses in humans.. Pharmacol Biochem Behav.

[22] Szeto GL, Brice AK, Yang HC, Barber SA, Siliciano RF (2010). Minocycline attenuates HIV infection and reactivation by suppressing cellular activation in human CD4^+^ T cells.. J Infect Dis.

[23] Ratai EM, Bombardier JP, Joo CG, Annamalai L, Burdo TH (2010). Proton magnetic resonance spectroscopy reveals neuroprotection by oral minocycline in a nonhuman primate model of accelerated NeuroAIDS.. PLoS One.

[24] Nishimura T, Ishima T, Iyo M, Hashimoto K (2008). Potentiation of nerve growth factor-induced neurite outgrowth by fluvoxamine: role of sigma-1 receptors, IP3 receptors and cellular signaling pathways.. PLoS ONE.

[25] Ishima T, Nishimura T, Iyo M, Hashimoto K (2008). Potentiation of nerve growth factor-induced neurite outgrowth in PC12 cells by donepezil: role of sigma-1 receptors and IP_3_ receptors.. Prog Neuropsychopharmacol Biol Psychiatry.

[26] Huang EJ, Reichardt LF (2001). Trk receptors: roles in neuronal signal transduction.. Annu Rev Biochem.

[27] Chao MV (2003). Neurotrophins and their receptors: a convergence point for many signaling pathways.. Nature Rev Neurosci.

[28] Read DE, Gorman AM (2009). Involvement of Akt in neurite outgrowth.. Cell Mol Life Sci.

[29] Gafni J, Munsch JA, Lam TH, Catlin MC, Costa LG (1997). Xestospongins: potent membrane permeable blockers of the inositol 1,4,5-trisphosphate receptor.. Neuron.

[30] Alano CC, Kauppinen TM, Valls AV, Swanson RA (2006). Minocycline inhibits poly (ADP-ribose) polymerase-1 at nanomolar concentrations.. Proc Natl Acad Sci USA.

[31] Song Y, Wei EQ, Zhang WP, Ge QF, Liu JR (2006). Minocycline protects PC12 cells against NMDA-induced injury via inhibiting 5-lipoxygenase activation.. Brain Res.

[32] Rogers GW, Komar AA, Merrick WC (2002). eIF4A: the godfather of the DEAD box helicases.. Prog Nucleic Acid Res Mol Biol.

[33] Kapp LD, Lorsch JR (2004). The molecular mechanisms of eukaryotic translation.. Annu Rev Biochem.

[34] Hernández G, Vazquez-Pianzola P (2005). Functional diversity of the eukaryotic translation initiation factors belonging to eIF4 families.. Mech Dev.

[35] Sofroniew MV, Howe CL, Mobley WC (2001). Nerve growth factor signaling, neuroprotection, and neural repair.. Annu Rev Neurosci.

[36] Stephens RM, Loeb DM, Copeland TD, Pawson T, Greene LA (1994). Trk receptors use redundant signal transduction pathways involving SHC and PLC-gamma 1 to mediate NGF responses.. Neuron.

[37] Kimura K, Hattori S, Kabuyama Y, Shizawa Y, Takayanagi J (1994). Neurite outgrowth of PC12 cells is suppressed by wortmannin, a specific inhibitor of phosphatidylinositol 3-kinase.. J Biol Chem.

[38] Costa-Mattioli M, Sossin WS, Klann E, Sonenberg N (2009). Translational control of long-lasting synaptic plasticity and memory.. Neuron.

[39] Berman RM, Cappiello A, Anand A, Oren DA, Heninger GR (2000). Antidepressant effects of ketamine in depressed patients.. Biol Psychiatry.

[40] Zarate CA, Singh JB, Carlson PJ, Brutsche NE, Ameli R (2006). A randomized trial of an N-methyl-D-aspartate antagonist in treatment-resistant major depression.. Arch Gen Psychiatry.

[41] Sanacora G, Zarate CA, Krystal JH, Manji HK (2008). Targeting the glutamatergic system to develop novel, improved therapeutics for mood disorders.. Nature Rev Drug Discov.

[42] Hashimoto K (2009). Emerging role of glutamate in the pathophysiology of major depressive disorder.. Brain Res Rev.

[43] Hashimoto K (2010). The role of glutamate on the action of antidepressants.. Prog Neuropsychopharmacol Biol Psychiatry in press.

[44] Li N, Lee B, Liu RJ, Banasr M, Dwyer JM (2010). mTOR-dependent synapse formation underlies the rapid antidepressant effects of NMDA antagonists.. Science.

[45] Zeng M, Zhou JN (2008). Roles of autophagy and mTOR signaling in neuronal differentiation of mouse neuroblastoma cells.. Cell Signal.

[46] Hoeffer CA, Klann E (2009). mTOR signaling: At the crossroads of plasticity, memory and disease.. Trends Neurosci.

[47] Levine J, Cholestoy A, Zimmerman J (1996). Possible antidepressant effect of minocycline.. Am J Psychiatry.

[48] Pae CU, Marks DM, Han C, Patkar AA (2008). Does minocycline have antidepressant effect?. Biomed Pharmacother.

[49] Henley J, Poo MM (2004). Guiding neuronal growth cones using Ca^2+^ signals.. Trends Cell Biol.

[50] Blosover SR (2005). Calcium signaling in growth cone migration.. Cell Calcium.

[51] Takei K, Shin RM, Inoue T, Kato K, Mikoshiba K (1998). Regulation of nerve growth mediated by inositol 1,4,5-trisphosphate receptors in growth cones.. Science.

[52] Minase T, Ishima T, Itoh K, Hashimoto K (2010). Potentiation of nerve growth factor-induced neurite outgrowth by the ROCK inhibitor Y-27632: a possible role of IP_3_ receptors.. Eur J Pharmacol.

[53] Jackson RJ, Hellen CUT, Pestova TV (2010). The mechanism of eukaryotic translation initiation and principles of its regulation.. Nature Rev Mol Cell Biol.

[54] Fukao A, Sasano Y, Imataka H, Inoue K, Sakamoto H (2009). The ELAV protein HuD stimulates cap-dependent translation in a Poly(A)- and eIF4A-dependent manner.. Mol Cell.

[55] Chen CY, Shyu AB (2009). HuD stimulates translation via eIF4A.. Mol Cell.

[56] Sonenberg N, Hinnebusch AG (2009). Regulation of translation initiation in eukaryotes: mechanisms and biological targets.. Cell.

[57] Abbott AM, Proud CG (2004). Translation factors: in sickness and in health.. Trends Biochem Sci.

[58] Scheper GC, van der Knaap MS, Proud CG (2007). Translation matters: protein synthesis defects in inherited disease.. Nature Rev Genet.

[59] Ferrer I (2002). Differential expression of phosphorylated translation initiation factor 2 alpha in Alzheimer's disease and Creutzfeldt-Jakob's disease.. Neuropathol Appl Neurobiol.

[60] Bruno MA, Leon WC, Fragoso G, Mushynski WE, Almazan G (2009). Amyloid b-induced nerve growth factor dysmetabolism in Alzheimer disease.. J Neuropathol Exp Neurol.

