# Correction: A Novel Target of Action of Minocycline in NGF-Induced Neurite Outgrowth in PC12 Cells: Translation Initiation Factor eIF4AI

**DOI:** 10.1371/annotation/afc0a9a2-01c0-4e58-8d69-e0ed4ff953fa

**Published:** 2010-12-03

**Authors:** Kenji Hashimoto, Tamaki Ishima

The word "Initiation" was incorrectly entered as "Initiator." The correct title is: "A Novel Target of Action of Minocycline in NGF-Induced Neurite Outgrowth in PC12 Cells: Translation Initiation Factor eIF4AI". The correct citation is: Hashimoto K, Ishima T (2010) A Novel Target of Action of Minocycline in NGF-Induced Neurite Outgrowth in PC12 Cells: Translation Initiator Factor eIF4AI. PLoS ONE 5(11): e15430. doi:10.1371/journal.pone.0015430

