# Supplementary figures and images for: A Novel Target of Action of Minocycline in NGF-Induced Neurite Outgrowth in PC12 Cells: Translation Initiation Factor eIF4AI
Source: PLoS One. 2010 Nov 8;5(11):e15430. doi: 10.1371/journal.pone.0015430 (PMC2975708; doi:10.1371/journal.pone.0015430)

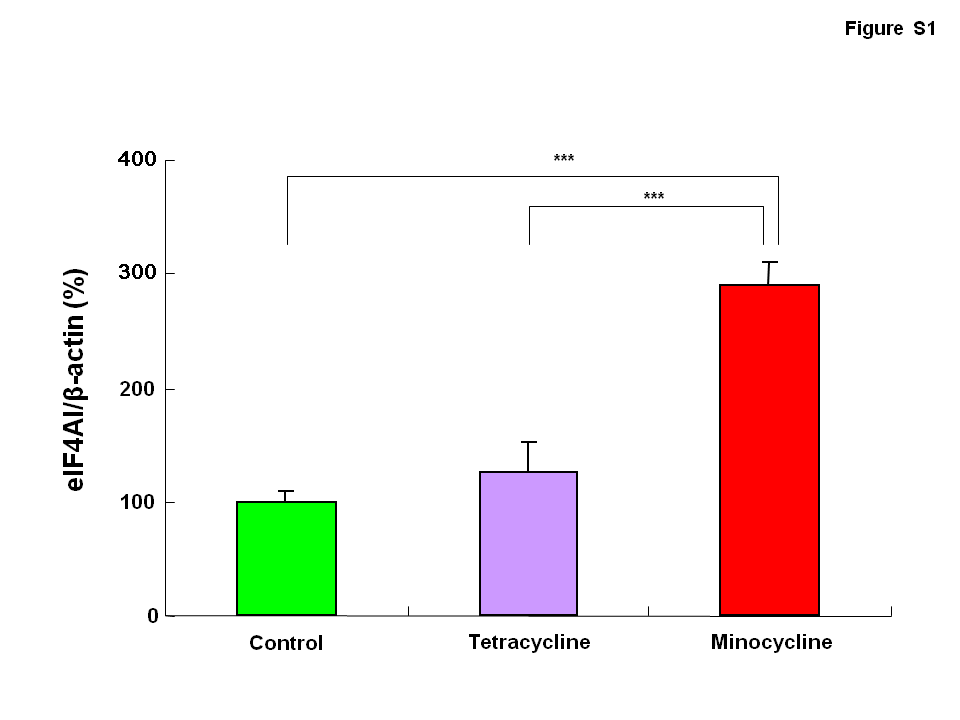

Supplement: Figure S1 — Effects of minocycline and tetracycline on eIF4AI levels in PC12 cells PC12 cells were treated with control (NGF (2.5 ng/ml)), minocycline (30 µM) or tetracycline (30 µM) for 5 days. Then cells were washed with PBS, and lysed in Laemmli lysis buffer. Western blot analysis was performed using rabbit anti-eIF4AI antibody (1∶250, ab31217, Abcam, Cambridge, UK). The levels of eIF4AI protein in PC12 cells were significantly increased by treatment with minocycline (30 µM), but not tetracycline (30 µM). The data show the mean ± SEM (n = 8). ***p<0.001 as compared with control (NGF (2.5 ng/ml) alone) group. (TIF) [file pone.0015430.s001.tif]

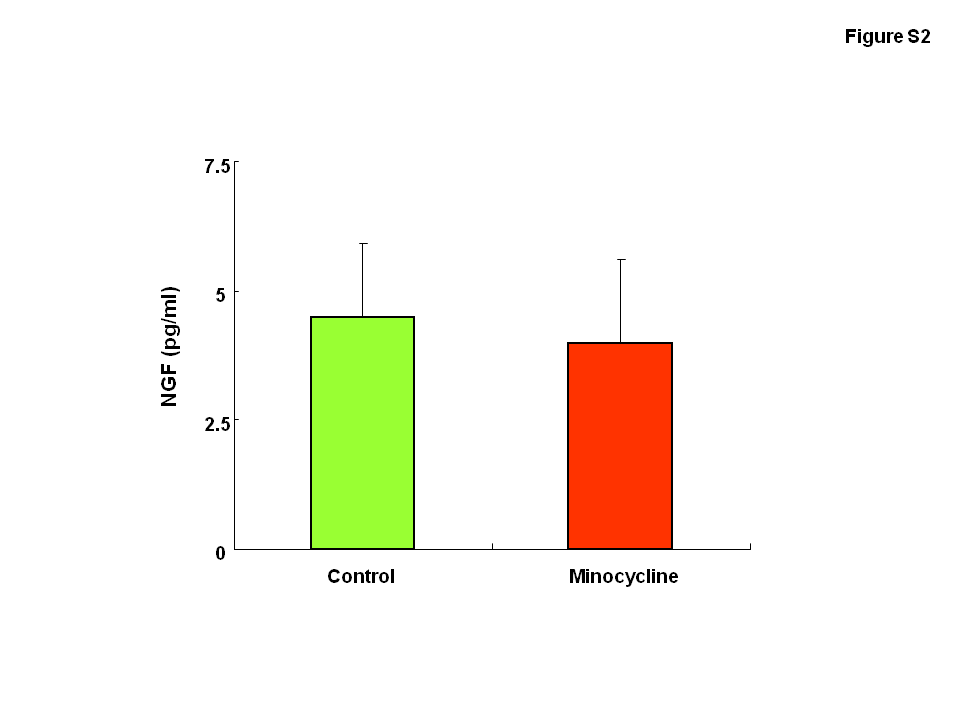

Supplement: Figure S2 — Effects of minocycline on NGF levels in PC12 cells PC12 cells were treated with control (NGF (2.5 ng/ml)) or minocycline (30 µM) for 5 days. The levels of NGF in the culture medium were measured using NGF Emax Immunoassay system (Promega, Madison, WI). The levels of NGF in the culture medium of PC12 cells were not altered by treatment with minocycline (30 µM). The data show the mean ± SEM (n = 6). (DOC) [file pone.0015430.s002.tif]
